# Emmet’s Operation Followed by Pregnancy in Three Months

**Published:** 1885-10

**Authors:** George W. Christian

**Affiliations:** Burnet, Texas


### EMMET'S OPERATION FOLLOWED BY PREGNANCY IN LESS THAN THREE MONTHS.

*By George W. Christian, M. D., Burnet, Texas.*

For Daniel's Texas Medical Journal.

MRS. L. BARR, aged 28, mother of three children, the youngest 4 years, had been in bad health since birth of last child; was having histero-epilepsy, which was attributed to uterine disease, as she had all rational symptoms. Digital and bi-manual examination disclosed a bi-lateral laceration of half an inch or more, with subinvolution, retroflexion and eversion. After proper preparatory treatment for about six weeks, I did trachelorrhaphy on August 7, 1884. On the next day she had a chill, which was followed by high fever; it proved, however, to be a purely remittent form of fever, and no cellular or peritoneal inflammation followed. She continued quite ill for seven days, and the stitches were not removed until the eighth day. Union was perfect. The womb measured half an inch less in depth than on day of operation.

Reposition, and a Smith-Hodge pessary was introduced. A few days after her getting up, her health rapidly improved. Convulsions ceased, and she continued in good health. After conception, three months, pessary was removed. On July 21, 1885, delivered her of a son. The labor was quite an easy one. Could not detect that the womb had ever been subjected to an operation. No laceration occurred either of cervix or perineum at this labor.

